# Reversing Persistent PTEN Activation after Traumatic Brain Injury Fuels Long‐Term Axonal Regeneration via Akt/mTORC1 Signaling Cascade

**DOI:** 10.1002/advs.202410136

**Published:** 2024-12-16

**Authors:** Ziyu Shi, Leilei Mao, Shuning Chen, Zhuoying Du, Jiakun Xiang, Minghong Shi, Yana Wang, Yuqing Wang, Xingdong Chen, Zhi‐Xiang Xu, Yanqin Gao

**Affiliations:** ^1^ State Key Laboratory of Medical Neurobiology MOE Frontiers Center for Brain Science and Institutes of Brain Science Fudan University Shanghai China; ^2^ Department of Neurosurgery Huashan Hospital Fudan University Shanghai China

**Keywords:** axon regeneration, cortical remapping, mTORC1, PTEN, TBI

## Abstract

Traumatic brain injury (TBI) often leads to enduring axonal damage and persistent neurological deficits. While PTEN's role in neuronal growth is recognized, its long‐term activation changes post‐TBI and its effects on sensory‐motor circuits are not well understood. Here, it is demonstrated that the neuronal knockout of PTEN (PTEN‐nKO) significantly enhances both structural and functional recovery over the long term after TBI. Importantly, in vivo, DTI‐MRI revealed that PTEN‐nKO promotes white matter repair post‐TBI. Additionally, calcium imaging and electromyographic recordings indicated that PTEN‐nKO facilitates cortical remapping and restores sensory‐motor pathways. Mechanistically, PTEN negatively regulates the Akt/mTOR pathway by inhibiting Akt, thereby suppressing mTOR. Raptor is a key component of mTORC1 and its suppression impedes axonal regeneration. The restoration of white matter integrity and the improvements in neural function observed in PTEN‐nKO TBI‐treated mice are reversed by a PTEN/Raptor double knockout (PTEN/Raptor D‐nKO), suggesting that mTORC1 acts as a key mediator. These findings highlight persistent alterations in the PTEN/Akt/mTORC1 axis are critical for neural circuit remodeling and cortical remapping post‐TBI, offering new insights into TBI pathophysiology and potential therapeutic targets.

## Graphical abstract

PTEN remains persistently activated long‐term after TBI, leading to inhibited axonal regeneration and impaired neurofunction (yellow‐shadow). PTEN‐nKO promotes axonal regeneration and cortical remapping through activation of the Akt/mTOR signaling pathway, thereby improving TBI outcomes (white shadow). However, neuronal PTEN/Raptor double knockout reverses the reparative effects of PTEN‐nKO, primarily by blocking the Akt/mTORC1 axis (orange‐shadow).

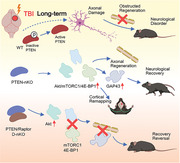

## 1 Introduction

Traumatic brain injury (TBI) exhibits the highest incidence among all neurological disorders, imposing a significant public health challenge. Patients with moderate‐to‐severe TBI commonly experience persistent cognitive, emotional, and functional issues that compromise their independence and quality of life.^[^ ^1^, ^2^, ^3^ ^]^ Previous research has primarily focused on gray matter injuries, recent studies indicate a strong correlation between white matter damage and declines in cognitive and sensorimotor functions following TBI.^[^ ^4^, ^5^ ^]^ Diffuse axonal injury, a prominent white matter pathological outcome of TBI, is characterized by the dynamic deformation of elongated white matter tracts, leading to gradual axonal impairment, delayed degeneration, and deterioration of synaptic structure and function.^[^ ^6^, ^7^ ^]^ This injury disrupts the ascending sensory tracts and the descending motor corticospinal tract (CST), linking the sensorimotor regions of the cerebral cortex to the spinal cord, resulting in sensory and motor deficits that may be partially mitigated through the restoration of these connections.^[^ ^8^ ^]^ Therefore, enhancing synaptic plasticity and promoting axonal regeneration are hypothesized to potentially improve TBI outcomes.^[^ ^9^ ^]^

Following acute central nervous system injuries such as stroke or TBI, the brain undergoes a period of elevated neuroplasticity.^[^ ^10^ ^]^ This results in the formation of new axons and synapses, which facilitates the recovery of sensorimotor functions through cortical remapping.^[^ ^11^ ^]^ Studies have demonstrated that motor cortex damage in primates leads to long‐distance axons sprouting,^[^ ^12^ ^]^ and enhanced cortical remapping is linked to restored sensorimotor function in mice after TBI.^[^ ^13^ ^]^ However, effective approaches to promote axonal regeneration following TBI remain limited. The neuronal intrinsic growth‐promoting factor, mTOR, plays a critical role in axonal growth.^[^ ^14^ ^]^ In a spinal cord injury model, mTOR activation has been shown to be crucial for programming neuronal intrinsic growth and promoting axon regeneration.^[^ ^15^ ^]^ This suggests that mTOR is involved in the regulation of axon repair. mTOR primarily functions by assembling intracellular proteins to form the mTOR complex (mTORC), subsequently initiating protein synthesis via the phosphorylation of eIF4E binding protein 1 (4E‐BP1).^[^ ^16^ ^]^

PTEN (Phosphatase and tensin homolog deleted on chromosome ten) is widely expressed in the mouse brain, particularly in neurons. Initially identified as a tumor suppressor gene, PTEN plays a crucial role in regulating various cellular processes such as cell cycle progression, migration, regeneration, metabolism, and autophagy.^[^ ^17^ ^]^ PTEN inhibitors have shown efficacy in reducing acute brain injury in rodent models of TBI.^[^ ^18^, ^19^ ^]^ However, due to the limited effectiveness of therapies in the acute phase, it is important to focus on nerve repair, particularly the regeneration and repair of axonal synapses in the chronic phase of TBI.^[^ ^20^ ^]^ PTEN, as an upstream negative regulator of the Akt signaling pathway by inhibiting Akt, directly inhibits Akt and in turn, indirectly suppresses mTOR phosphorylation. Subsequently, mTOR recruits mTOR complex 1 (mTORC1), specifically incorporating the Raptor protein in vivo. mTORC1 then promotes the synthesis of proteins involved in axonal regeneration, such as GAP43, by phosphorylating p70S6 kinase 1 (S6K1) and eIF4E binding protein 1 (4E‐BP1).^[^ ^21^, ^22^ ^]^ Given that activated mTOR plays a critical role in axonal repair, it is hypothesized that PTEN may influence neural axonal regeneration through its effects on the AKT/mTOR/mTORC1 pathway.^[^ ^14^ ^]^ A study using neurosphere cultures derived from PTEN‐deficient mice has revealed increased proliferation and self‐renewal capabilities.^[^ ^23^ ^]^ Furthermore, PTEN‐knockdown has been shown to increase cortical mTOR activity and enhance corticospinal axon regeneration in mice after spinal cord injury (SCI).^[^ ^24^ ^]^ This suggests that inhibiting PTEN activity may have the capability to enhance neurogenesis.^[^ ^14^ ^]^ However, the precise relationship between PTEN and axonal regeneration following TBI has yet to be determined.

Our study revealed that PTEN exhibited sustained activation for 35 days following TBI, promoting us to investigate its role in axonal regeneration during the chronic phase post‐TBI. Utilizing neuron‐specific PTEN knockout (PTEN‐nKO) transgenic mice, our study demonstrated that PTEN‐nKO promotes compensatory axon growth and synaptic remodeling in the chronic stage of TBI. Mechanistically, PTEN‐nKO sustains AKT activation, which in turn enhances axonal repair by activating the mTORC1/4E‐BP1 pathway and increasing GAP43 expression. Furthermore, PTEN‐nKO not only enhances axonal repair in the central nervous system but also contributes to the restoration of both ascending sensory and descending motor tracts through cortical remapping. Our study provides significant insights into the role of neuronal PTEN in TBI recovery and highlights a potential novel therapeutic target for acute brain injuries.

## 2 Result

### 2.1 PTEN Activation Persists After TBI in Both Human Patients and Mouse Model

**Figure 1:**
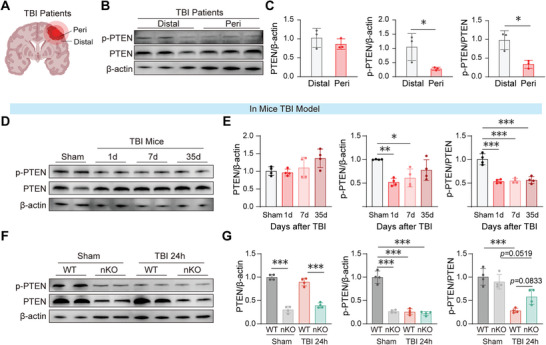
Activation of PTEN in neurons following TBI. A) Schematic diagram of tissue specimen collection around the injury site in a patient with acute cerebral contusion. B,C) Representative images and statistical plots of a Western blot showing levels of p‐PTEN and PTEN in peri‐injury and distal tissues of patients. *n* = 3/group. D,E) Representative images and statistical plots of Western blot showing p‐PTEN and PTEN levels in peri‐injury tissues on days 1, 7, and 35 post‐TBI. *n* = 4/group. F,G) Representative images and statistical plots of a Western blot showing relative protein content of p‐PTEN and PTEN in peri‐injury tissues of WT versus nKO mice post‐TBI. *n* = 4/group. All data are presented as means±SD. Data were analyzed using unpaired two‐tail Student's *t*‐test C), or one‐way ANOVA followed by Bonferroni's post hoc test E,G). *p < 0.05, **p < 0.01, ***p < 0.001, ns: no significance, as indicated.

Under physiological conditions, PTEN is predominantly localized in the cytoplasm in an inactivated, phosphorylated state.^[^ ^25^ ^]^ Following neural injury, PTEN becomes dephosphorylated and activated, thereby exerting a negative regulatory effect on the Akt pathway.^[^ ^26^ ^]^ To investigate the temporal dynamics of PTEN activation following TBI, we first assessed PTEN activation in the brain tissues from patients with acute brain contusion and laceration, a prevalent form of TBI in clinical practice.^[^ ^27^ ^]^ We collected tissue samples during emergency neurosurgical procedures (Figure S1A, Supporting Information) and analyzed PTEN activation in areas near the injury site (Peri) and in distal areas resembling healthy brain tissue (Distal) (**Figure** **1**A). Consistent with our hypothesis, the overall PTEN expression levels did not change between the Peri and Distal regions (Figure 1B,C left panel). However, both the expression of phosphorylated PTEN (p‐PTEN) and the phosphorylation ratio of PTEN significantly decreased (Figure 1B,C right two panels), indicating pronounced PTEN activation in the brain tissues damaged by TBI.

To further investigate the temporal dynamics of PTEN activation following TBI, we utilized a controlled cortical impact (CCI) model to simulate moderate to severe TBI in mice.^[^ ^18^ ^]^ We quantified the levels of p‐PTEN and total PTEN in peri‐injury tissues on day 1, 7, and 35 postinjury and found that the overall expression levels of PTEN remained consistent from day 1 to day 35 post‐TBI, albeit with a slight upward trend (Figure 1D,E, right panel). Notably, during the acute and subacute phases from day 1 to 7 post‐TBI, p‐PTEN levels were significantly reduced to ≈50% of those in sham‐operated controls (Figure 1D,E middle panel). Moreover, throughout the entire period from day 1 to 35 post‐TBI, the phosphorylation levels of PTEN were persistently and significantly decreased, maintaining at roughly 50% of sham levels (Figure 1D,E right panel). These results demonstrated a sustained activation of PTEN for up to 35 days following TBI, prompting us to hypothesize that PTEN plays a crucial role in the long‐term neurological response to TBI.

To examine the sustained impact of PTEN on neuronal recovery post‐TBI, we generated PTEN^flox/flox^MAP2^CreERT2^ mice (PTEN‐nKO) by crossing PTEN^flox/flox^ mice with MAP2^CreERT2^ mice (Figure S1B, Supporting Information). In these PTEN‐nKO mice, PTEN knockout is initiated exclusively post‐tamoxifen injection, minimizing the potential developmental impacts of PTEN deletion on the nervous system. To confirm the effectiveness of the knockout, western blot analysis revealed that 24 h post‐TBI, PTEN expression in PTEN‐nKO mice was reduced by 69.6% compared to wild‐type (WT) mice (Figure 1F,G left panel). Additionally, the reduction of PTEN phosphorylation observed following TBI remained unaltered in PTEN‐nKO mice, while PTEN phosphorylation was significantly decreased in WT TBI mice (Figure 1F,G right two panels).

### 2.2 PTEN‐nKO Ameliorates Long‐Term TBI Neurofunctional Outcomes

**Figure 2:**
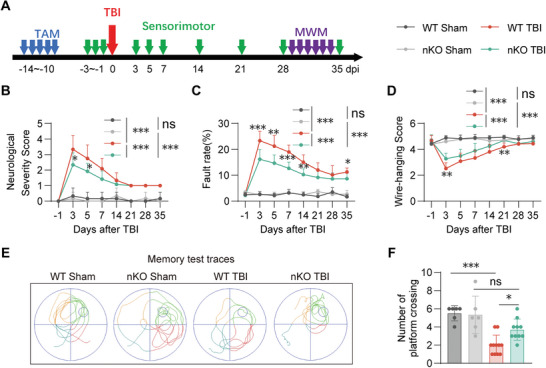
PTEN‐nKO improves long‐term sensorimotor and memory outcomes post‐TBI. A) Experimental design for behavioral tests. B‐D) Statistical chart of neurological function scores B), grid walking test C), and the wire‐hanging test D), *n* = 6/6/12/12 (WT Sham/nKO Sham/WT TBI/nKO TBI). E) Representative swimming paths during the memory phases of the Morris water maze test (34 days post‐TBI). F) Statistical plots of the number of platform crossings during the test phase (34 days post‐TBI). *n* = 6/6/11/10 (WT Sham/nKO Sham/WT TBI/nKO TBI). All data are presented as means±SD. Data were analyzed using two‐way ANOVA B–D) or one‐way ANOVA F) followed by Bonferroni's post hoc test. *p < 0.05, **p < 0.01, ***p < 0.001, ns: no significaance, as indicated.

Clinically, recovery following TBI is commonly assessed using neurobehavioral evaluations and imaging techniques.^[^ ^28^, ^29^ ^]^ In this study, we conducted a bunch of neurobehavioral tests to investigate the impact of PTEN‐nKO on neurological recovery during days 3–35 post‐TBI (**Figure** **2**A). We extensively evaluated the restoration of sensorimotor functions in mice by measuring somatic asymmetry using the Neurological Severity Score (NSS), assessing spatial perception and motor control via the grid‐walking test, and evaluating upper limb muscle strength through the wire‐hanging test. The results indicated that mice in the TBI group exhibited significant long‐term sensorimotor impairments post‐TBI, whereas PTEN‐nKO substantially mitigated these sensorimotor deficits (Figure 2B–D). Furthermore, results from the Morris water maze test, used to assess spatial learning and memory, revealed that although PTEN‐nKO did not ameliorate TBI‐induced learning deficits (Figure S2A–C, Supporting Information), it significantly enhanced recovery from TBI‐induced memory impairments (Figure 2E,F). Additionally, PTEN‐nKO markedly reversed the reduction in neuronal counts observed in the regions surrounding the lesions (Figure S2D–F, Supporting Information) and substantially alleviated long‐term tissue damage post‐TBI (Figure S2G,H, Supporting Information). Taken together, these data suggested that PTEN‐nKO may improve the long‐term outcome following TBI.

### 2.3 PTEN‐nKO Enhances the Integrity of Synapses and Axons Following TBI

**Figure 3:**
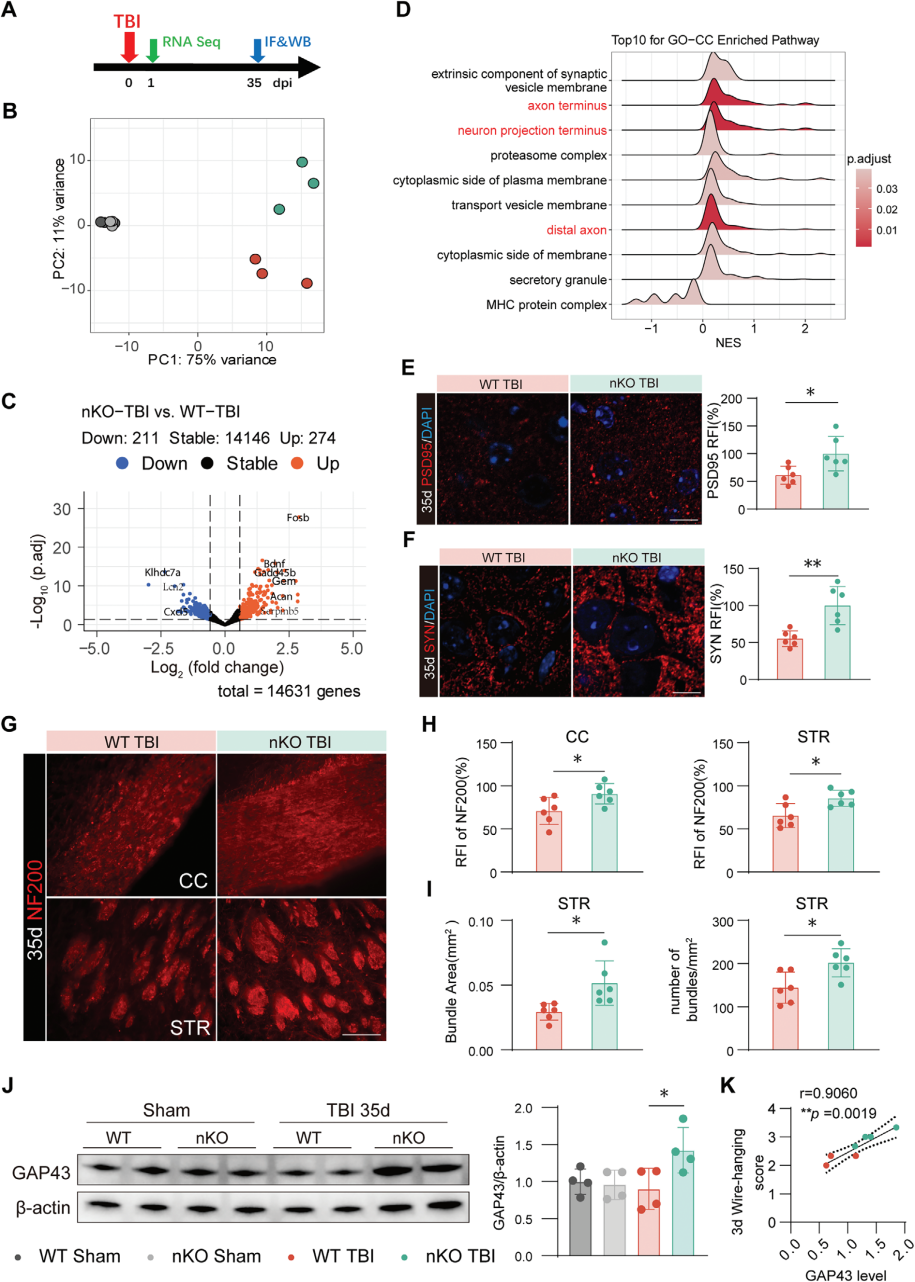
PTEN‐nKO may promote synaptic and axonal regeneration and repair after TBI. A) Experimental design for the RNA‐seq and long‐term axonal regeneration. B) Principal component analysis plot between the four groups. *n* = 3/group. C) Volcano plot of differential expression genes for nKO TBI versus WT TBI. D) Pathways enriched by KEGG analysis of differentially expressed genes in the nKO TBI group relative to the WT TBI group. E) Representative images and statistical plots of PSD95 (red) and DAPI (blue) immunofluorescence in the CTX of peri‐injury area 35 days post‐TBI, bar: 10 µm. F) Representative images and statistical plots of synaptophysin (red) and DAPI (blue) immunofluorescence in the peri‐injury zone of the CTX at 35 days post‐TBI, bar: 10 µm. G) Representative images of NF200 immunofluorescence staining in the CC and STR of peri‐injury area, bar: 100 µm. H) Statistical plots of NF200 relative fluorescence intensity in the CC and STR 35 days post‐TBI. I) Statistical plots of the fiber bundles in the STR of the peri‐injury area 35 days post‐TBI. J) Representative images and statistical plots of the GAP43 western blot 35 days post‐TBI. K) Correlation analysis between the wire‐hanging score and the GAP43 level. r: Spearman correlation coefficient. All data are presented as means±SD. Data were analyzed using unpaired two‐tail Student's *t*‐test (E‐I, *n* = 6/group), one‐way ANOVA followed by Bonferroni's post hoc test (J, *n* = 4 per group), or Spearman correlation test (K, *n* = 4 per group). *p < 0.05, **p < 0.01, as indicated.

To thoroughly explore the potential protective mechanisms conferred by PTEN deficiency in neurons post‐TBI, we performed bulk RNA‐seq of peri‐injured tissues collected from four groups of mice one day following TBI (**Figure** **3**A). Differential gene expression analysis between the nKO TBI and WT TBI groups revealed significant differences. Principal component analysis (PCA) revealed that the transcriptomes of the two Sham groups were highly similar, indicating that PTEN knockdown in adulthood does not significantly affect the transcriptome under normal conditions. However, a significant difference was observed between the two TBI groups, suggesting altered gene expression profiles post‐TBI (Figure 3B). Further analysis showed no differentially expressed genes between WT Sham and nKO Sham (Figure S3A, Supporting Information), aligning with the PCA results. In contrast, WT TBI resulted in the upregulation of 924 genes and the downregulation of 223 genes (Figure S3B, Supporting Information). Meanwhile, nKO TBI led to the upregulation of 274 genes and downregulation of 211 genes (Figure 3C). Subsequently, we employed the Kyoto Encyclopedia of Genes and Genomes (KEGG) for enrichment analysis using the Gene Set Enrichment Analysis (GSEA) method. Our study found that axon‐related pathways were significantly enriched in the nKO TBI group, with axon terminus being the most significant, followed closely by neuron projection terminus and distal (Figure 3D), suggesting that PTEN‐nKO may promote the long‐term repair of axons and synapses.

Following TBI, white matter fibers surrounding the injury site deteriorate, and diffuse axonal damage extends into more distal white matter regions, which significantly impacts brain function.^[^ ^30^, ^31^ ^]^ Given the RNA‐seq results, we aimed to further investigate the potential protective role of PTEN‐nKO in short‐ or long‐term structural and functional recovery of white matter fibers post‐TBI. The relative fluorescence intensities (RFI) of the presynaptic protein (synaptophysin, Syn) and the postsynaptic density protein 95 (PSD95) were markedly enhanced in the peri‐injury region of the nKO TBI group 35 days post‐TBI compared to the WT TBI group (Figure 3E,F). This observation suggested that PTEN‐nKO provided significant protective effects on synaptic proteins in the long term. Notably, this protective effect, particularly on the presynaptic protein SYN, was also evidenced in the acute phase. However, the degree of protection observed in the acute phase was less pronounced compared to the long‐term effects. This was supported by our SYN staining results at 1 and 7 days post‐TBI, which showed a similar but less substantial protective effect of PTEN‐nKO on synaptic protein levels during the early phase (Figure S4A, Supporting Information).

In our assessment of white matter axon organization, myelin density, indicated by the relative fluorescence intensity of myelin basic protein (MBP), showed no significant differences between the nKO TBI and WT TBI groups 35 days post‐TBI (Figure S4B–E, Supporting Information). However, the corpus callosum (CC) thickness was notably greater in the PTEN‐nKO TBI mice compared to the WT TBI mice (Figure S4F, Supporting Information), suggesting PTEN‐nKO may counteract TBI‐induced white matter atrophy. Furthermore, when labeling nerve axons with neurofilament 200 (NF200) 35 days post‐TBI, we observed significantly higher fluorescence intensities in the CC and striatum (STR) areas surrounding the injury site in nKO TBI mice compared to the WT TBI mice (Figure 3G,H). Moreover, the area and number of NF200‐labeled nerve bundles in the STR were significantly increased in nKO TBI mice (Figure 3I). Additionally, growth‐associated protein 43 (GAP43), a membrane protein crucial for axon growth and synaptogenesis,^[^ ^32^ ^]^ was significantly more expressed in nKO TBI mice compared to WT TBI mice 35 days post‐TBI (Figure 3J). Interestingly, the expression levels of GAP43 also showed significant correlations with behavioral outcomes (Figure 3K).

Collectively, these findings suggested that PTEN‐nKO may alleviate long‐term structural damage in white matter after TBI by promoting axonal and synaptic integrity, thereby facilitating the recovery of motor functions.

### 2.4 PTEN‐nKO Exerts Protective Effects via Promoting the Long‐Term Regeneration and Repair of White Matter Axons

**Figure 4:**
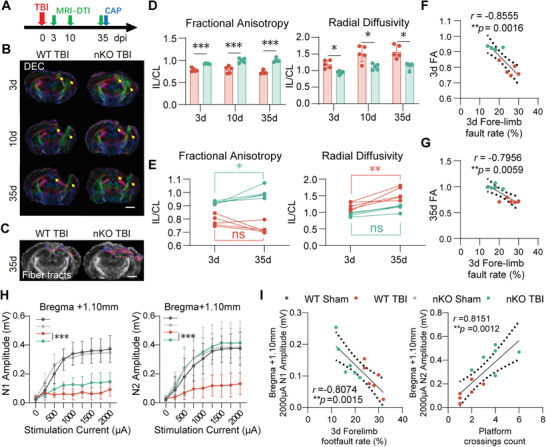
PTEN‐nKO promotes white matter structural and functional recovery in the long term after TBI. A) Experimental design for the MRI‐DTI and CAP. B) DEC (direction encoded color) map presented was used to visualize in vivo DTI on day 3, 10, and 35 days post‐TBI, scale bar: 3 mm. C) Representative images for reconstruction of the CC white matter fiber bundle in the injury side 35 days post‐TBI, the different colors indicating fiber direction, scale bar: 3 mm. D) Statistical plots showing changes in FA (fractional anisotropy, left panel) and RD (radial diffusivity, right panel) in the CC area on day 3, 10, and 35 post‐TBI, *n* = 5/group. E) Changes in FA (left panel) and RD (right panel) from 3 to 35 days post‐TBI, *n* = 5/group. F) Spearman correlation analysis between forelimb misstep rate and the 3‐day FA index in the grid walking experiment, *n* = 5/group. G) Spearman correlation analysis of forelimb misstep rate and 3‐day FA metrics in the grid walking experiment, *n* = 5/group. H) Statistical charts showing CAPs N1 and N2 data for bregma +1.1 mm 35 days post‐TBI, *n* = 6/group. I) Spearman correlation analysis of the forelimb misstep rate 3 days post‐TBI with N1 amplitude at a stimulus intensity of 2000 µA (left) 35 days post‐TBI, and the number of times crossing the platform in the Morris water maze 34 days with N2 amplitude at a stimulus intensity of 2000 µA (right), respectively. r: Spearman correlation coefficient, *n* = 6/group. All data are presented as means±SD. Data were analyzed using two‐way ANOVA followed by Bonferroni post hoc test *p < 0.05, **p < 0.01, ***p < 0.001, as indicated.

To further investigate white matter integrity post‐TBI, we employed Diffusion Tensor Imaging (DTI) on days 3, 10, and 35 (**Figure** **4**A).^[^ ^33^ ^]^ The 3D reconstruction of white matter fibers in the CC surrounding the injury revealed that PTEN‐nKO TBI mice exhibited a higher density of white matter fibers compared to the WT TBI mice (Figure 4B,C). Additionally, Fractional Anisotropy (FA, reflecting the fine structure of white matter) and Radial Diffusivity (RD, indicating demyelination or axonal swelling),^[^ ^34^, ^35^ ^]^ showed significant improvements in PTEN‐nKO TBI mice at all assessed time points (Figure 4D)

Interestingly, when comparing the FA and RD values between 3 and 35 days post‐TBI, we surprisingly observed a significant increase in FA values at 35 days compared to 3 days in nKO TBI mice, while there was no significant change in FA values over the same period in WT TBI mice (Figure 4E, left panel). Furthermore, RD values at 35 days were significantly higher than at 3 days in WT TBI mice, while nKO TBI mice showed no significant change in RD values during the same period (Figure 4E, right panel). These data were consistent with previous synaptic protein detection results (Figure 3E,F; Figure S4A, Supporting Information), indicating that PTEN‐nKO promotes the repair of white matter structure and mitigates the progression of axonal damage following TBI. Additionally, the improvement in FA was significantly correlated with reductions in foot‐fault rates, demonstrating the close relationship between white matter structural repair and behavioral recovery (Figure 4F,G).

To assess the functional integrity of white matter, we recorded compound action potentials (CAPs) in the CC (Bregma + 1.10 mm) 35 days after TBI.^[^ ^36^ ^]^ Faster action potentials (N1), indicative of myelinated fiber conductance, and slower action potentials (N2), indicative of unmyelinated fiber conductance, were analyzed (Figure S5, Supporting Information).^[^ ^28^ ^]^ Compared to mice in the WT TBI group, nKO TBI mice showed a significant recovery in the amplitudes of both N1 and N2, with a more pronounced recovery noted for N2 (Figure 4H). Furthermore, we found that the recovery of N1 and N2 was significantly correlated with improvements in sensorimotor and memory performance (Figure 4I). The CAPs results suggested that PTEN‐nKO enhances the functional recovery of damaged white matter, particularly improving the function of unmyelinated axons.

Taken together, our findings demonstrated that PTEN deficiency in neurons significantly promotes the long‐term structural recovery of white matter after TBI, and ultimately enhances white matter axonal function.

### 2.5 PTEN‐nKO Enhances Neural Remodeling and Cortical Remapping After TBI

**Figure 5:**
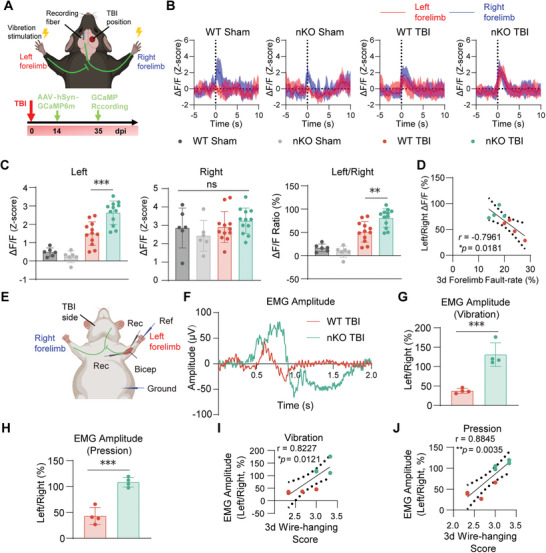
PTEN‐nKO promotes cortical remapping and neural remodeling after TBI. A) Schematic diagram illustrating the experimental design and methodology for fiber‐optic recording of calcium signal of cortical remapping. B) Representative recording of calcium signal changes in response to vibratory stimulation of both forelimbs, detected by fiber optics in the S1‐FL region 35 days post‐TBI. The time range is from 5s before to 10s after stimulation, with red line indicating the left forelimb with impaired function and blue line indicating the right forelimb with normal function. C) Statistical plots of calcium signal changes in response to vibration stimuli 35 days post‐TBI for the left limb (left panel), right limb (middle panel) and left/right ratio (right panel). Sham groups: *n* = 2 animals × 3 times per group; TBI groups: *n* = 4 animals × 3 times per group. D) Spearman correlation analysis of the ratio of left to right forelimb calcium signals 35 days post‐TBI with the forelimb misstep rate from the grid walking experiment 3 days post‐TBI, *n* = 4 per group. E) Schematic diagram of the setup for recording EMG signals. Rec: Recording Electrode, Ref: Reference Electrode, Ground: Grounding Electrode. F) Representative recording of the EMG amplitude under vibration stimulation. G,H) Quantitation of EMG signal amplitude under vibration G) or compression H) stimulation. *n* = 4 per group. I,J) Spearman correlation analysis of the EMG amplitude under vibration or compression stimulation with wire‐hanging scores 3 days post‐TBI, *n* = 4 per group, r: Spearman correlation coefficient. All data are presented as means±SD. Data were analyzed using one‐way ANOVA followed by Bonferroni post hoc test C) or unpaired two‐tail Student's *t*‐test G,H). *p < 0.05, **p < 0.01, ***p < 0.001, ns: no significance, as indicated.

During TBI rehabilitation, the recovery of neurological functions largely depends on effective neural circuit remodeling and cortical remapping.^[^ ^11^, ^37^ ^]^ The brain can activate undamaged neural circuits in the contralateral cortex to compensate for impaired neural functions.^[^ ^8^ ^]^ To test whether the long‐term recovery effect of PTEN‐nKO after TBI is associated with enhanced axonal regeneration and cortical remapping, we injected the AAV‐hSyn‐DIO‐GCaMP6s virus into the left primary somatosensory cortex forelimb region (S1‐FL) of mice 14 days post‐TBI.^[^ ^38^ ^]^ An optical fiber was then implanted to record calcium signals from sensory neurons, with stimulation applied to both limbs using vibration on day 35 post‐TBI (**Figure** **5**A). In both sham groups, stimulation of the left forepaw (injured side) did not elicit an increased homolateral cortical neuronal calcium signal, whereas stimulation of the right forepaw (uninjured side) significantly activated contralateral cortical neuronal calcium signal (Figure 5B; Figure S6, Supporting Information, left two panels). This observation confirms that under normal physiological conditions, the left S1‐FL region controls the right forepaw, and the right S1‐FL controls the left forepaw.^[^ ^13^, ^39^ ^]^ After TBI, which damaged the right cortex, the brain engaged the left cortex to compensate for the impaired control of the left forepaw, a process known as cortical remapping.^[^ ^11^, ^40^ ^]^ In TBI mice, stimulation of the left forepaw resulted in a significantly higher calcium signal compared to sham mice (Figure 5B; Figure S6, Supporting Information, right two panels). Further analysis revealed that the calcium signal in nKO TBI mice following left forepaw stimulation was significantly greater than that in WT TBI mice, indicating enhanced cortical remapping due to PTEN deletion. In contrast, stimulation of the right forepaw showed no significant differences in calcium signal among the four groups (Figure 5C). These results demonstrated that PTEN deficiency in neurons significantly enhances cortical remapping, which is closely correlated with behavioral improvements (Figure 5D).

Previously, we found that PTEN‐nKO improves upper limb muscle strength after TBI (Figure 2D) and promotes the recovery of sensory upstream pathways (Figure 5A–D). To further investigate the recovery of motor downstream pathways after TBI, we stimulated both forepaws of mice with vibration or pressure, and simultaneously recorded electromyographic (EMG) signals of the biceps brachii muscle (Figure 5E).^[^ ^41^ ^]^ The nKO TBI group exhibited significantly higher EMG signal amplitudes in the left forelimb (the impaired side) compared to the WT TBI group (Figure 5F–H). Additionally, the amplitude of the EMG signals was significantly and positively correlated with the scores of the wire‐hanging test (Figure 5I,J), suggesting that PTEN‐nKO facilitated the recovery of the motor downstream pathway.

Collectively, these findings confirmed that neuronal PTEN deletion promotes cortical remapping and neural circuit remodeling after TBI.

### 2.6 PTEN‐nKO Promotes Cortical Remapping by Activating the Akt/mTOR/4E‐BP1 Pathway

As previously noted, PTEN remains continuously activated after the onset of TBI (Figure 1D,E), and this sustained activation may suppress the Akt/mTOR signaling pathway, potentially leading to cellular damage.^[^ ^42^ ^]^ In spinal cord injury models, previous studies have demonstrated that knocking down PTEN significantly enhances the regeneration of corticospinal neurons.^[^ ^24^ ^]^ Based on these observations, we hypothesize that knockdown of PTEN may facilitate neural regeneration and functional recovery by sustaining activation of the Akt/mTOR pathway after TBI.

**Figure 6:**
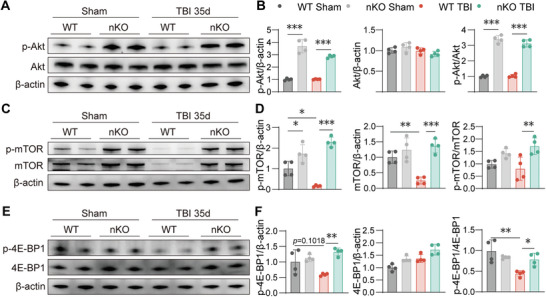
PTEN‐nKO activates the Akt/mTOR/4E‐BP1 signaling pathway in the long‐term after TBI. A,B) Representative images and statistical plots of western blot showing the expression of p‐Akt and Akt 35 days post‐TBI. C,D) Representative images and statistical plots of western blot showing the expression of p‐mTOR and mTOR expression 35 days post‐TBI. E,F) Representative images and statistical plots of western blot showing the expression of p‐4E‐BP1 and 4E‐BP135 days post‐TBI. All data are presented as means±SD, *n* = 4 per group. Data were analyzed using one‐way ANOVA followed by Bonferroni post hoc test. *p < 0.05, ^**^p < 0.01, ^***^p < 0.001, as indicated.

To test this hypothesis, we examined the activation of the Akt/mTOR signaling pathway in each group of mice 35 days after TBI. Western blot results showed that phosphorylation levels of Akt and mTOR were significantly elevated in both nKO Sham groups. Furthermore, the levels of p‐Akt and p‐mTOR in the nKO TBI group were significantly higher than those in the WT TBI group (**Figure** **6**A–D). Additionally, p‐4E‐BP1, a downstream target of mTORC1, was also notably elevated in the nKO TBI group (Figure 6E,F), while p‐4E‐BP1 was decreased in the WT TBI group. These data suggested that the Akt/mTOR/4E‐BP1 pathway is persistently activated in PTEN‐nKO TBI mice.

### 2.7 mTORC1 is Essential for Long‐Term Pro‐Repair Effects of PTEN‐nKO

mTOR forms two distinct complexes: mTOR complex 1 (mTORC1), which specifically contains the protein Raptor, and mTOR complex 2 (mTORC2), which contains the protein Rictor.^[^ ^43^, ^44^ ^]^ Activation of mTORC1 primarily facilitates cell growth through phosphorylating p70 S6 kinase 1 (S6K1) and eIF4E‐binding protein 1 (4E‐BP1), thereby promoting protein synthesis.^[^ ^45^ ^]^ Our previous findings showed that PTEN‐nKO exerts its effects by activating mTOR and its downstream target 4E‐BP1 (Figure 6E), with 4E‐BP1 primarily activated by mTORC1.

**Figure 7:**
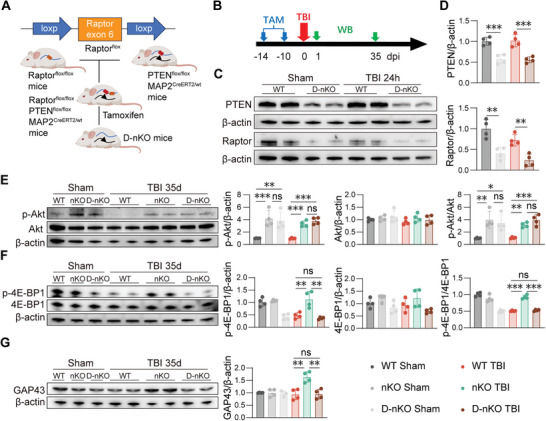
PTEN/Raptor D‐nKO reverses PTEN‐nKO‐induced mTORC1 activation after TBI. A) Schematic of the D‐nKO mice construction. B) The experimental design for the PTEN/Akt/mTOR pathway. C,D) Representative images and statistical plots of western blot showing the expression of PTEN and Raptor protein expression in D‐nKO group 1‐day post‐TBI. E) Representative image and statistical graphs of western blot showing the expression of p‐Akt and Akt 35 days post‐TBI. F) Representative image and statistical graphs of western blot showing the expression of p‐4E‐BP1 and 4E‐BP1 35 days post‐TBI. G) Representative images and statistical plots of western blot showing the GAP43 expression 35 days post‐TBI. All data are presented as means±SD, *n* = 4 per group. Data were analyzed using one‐way ANOVA followed by Bonferroni post hoc test. **p < 0.01, ***p < 0.001, ns: no significance, as indicated.

To further elucidate the core molecules through which PTEN‐nKO promotes long‐term repair after TBI, we generated Raptor^flox/flox^ mice and crossed them with PTEN‐nKO mice to obtain neuron‐specific Raptor/PTEN double knockout mice (D‐nKO) (**Figure** **7**A,B). The knockout effects were validated by Western blot analysis, which showed that the levels of PTEN and Raptor in the D‐nKO mice were reduced by over 50% compared to WT mice (Figure 7C,D). Subsequently, we assessed the molecular signaling pathways involved in the long‐term response to TBI, focusing specifically on upstream and downstream signaling alterations of mTORC1 in D‐nKO mice. Western blot results indicated that 35 days post‐TBI, both D‐nKO mice and nKO mice exhibited significantly higher levels of p‐Akt compared to WT in Sham or TBI mice, with no significant differences between D‐nKO TBI and nKO TBI mice (Figure 7E). These findings suggested that the double knockout of Raptor and PTEN did not influence Akt activation, the upstream of mTORC1, in PTEN‐nKO mice, indicating that Akt activation was consistently maintained in D‐nKO mice post‐TBI.

However, 35 days post‐TBI, nKO TBI mice showed significantly higher expression and phosphorylation levels of p‐4E‐BP1, a downstream molecular of mTOR signaling, compared to WT TBI and D‐nKO TBI mice, with no significant differences between WT TBI and D‐nKO TBI mice (Figure 7F). Additionally, GAP43 expression levels were significantly higher in nKO TBI mice than in WT TBI and D‐nKO TBI mice, with no significant differences between WT TBI and D‐nKO TBI mice (Figure 7G). These results indicate that the double knockout in D‐nKO TBI mice reverses the enhanced activation of the mTORC1 downstream molecule 4E‐BP1 and the upregulation of GAP43 mediated by PTEN‐nKO, suggesting that mTORC1 may play a critical role in the PTEN‐nKO mediated signaling pathway that promotes long‐term axonal recovery following TBI.

### 2.8 Raptor/PTEN‐D‐nKO Reverses the Neuroprotection of PTEN‐nKO on the Long‐Term Tissue Repair and Advanced Neurofunction Improvements After TBI

**Figure 8:**
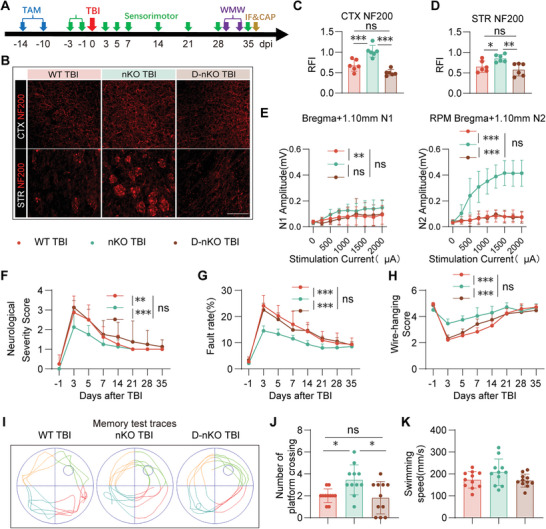
PTEN/Raptor D‐nKO reverses tissue and behavioral improvements induced by PTEN‐nKO after TBI. A) Experimental design for behavioral tests, IF and CAP. B–D) Representative images and statistical plots of immunofluorescence staining for NF200 (red) in the CTX C) and STR D) of the peri‐injury area 35 days post‐TBI. Scale bar: 100 µm. *n* = 6 per group. E) Statistical plots showing the N1 and N2 amplitudes for bregma+1.10 mm in CC on day 35 post‐TBI. *n* = 6/6/3 (WT TBI/nKO TBI/D‐nKO TBI). F–H) Sensorimotor deficits were evaluated before (‐1) and up to 35 days post‐TBI by neurological severity scores F), the grid walking test G), and the wire‐hanging test H), *n* = 8 per group. I) Representative swimming paths of three TBI groups during the memory phases of the Morris water maze test (34 days post‐TBI). J,K) Statistical plot of the number of platform crossings J) and swimming speeds K) during the memory test phase of the Morris water maze 34 days post‐TBI, *n* = 11/group. All data are presented as means±SD. Data were analyzed using one‐way ANOVA C,D, J,K) or two‐way ANOVA E–H) followed by Bonferroni post hoc test. *p < 0.05, **p < 0.01, ***p < 0.001, ns: no significance, as indicated.

We conducted a comprehensive assessment of the histological and behavioral outcomes to determine whether D‐nKO mice could structurally and functionally reverse the long‐term neuroprotection of PTEN‐nKO after TBI (**Figure** **8**A). We found that the relative fluorescence intensity of NF200 in D‐nKO TBI mice showed no significant difference compared to WT TBI mice, but it was significantly reduced compared to nKO TBI mice (Figure 8B–D). Similarly, the detection of CAPs in the CC area showed comparable results in the 3 TBI groups (Figure 8E), while the three sham groups (WT Sham, nKO Sham, and D‐nKO Sham) have no change (S7A‐B). Detailly, nKO TBI mice exhibited a significant recovery in N2 compared to the other two groups, while there was no significant difference in N2 between WT TBI mice and D‐nKO TBI mice (Figure 8E, left panel). Regarding N1, nKO TBI mice showed a significant recovery relative to WT TBI mice, whereas D‐nKO TBI mice showed no significant difference from WT TBI mice (Figure 8E, left panel). These data indicated that there was no significant improvement in the recovery of white matter structural integrity, especially the integrity of axonal structure and function, between the D‐nKO TBI mice and WT TBI mice, thus reversing the protective and reparative effects of PTEN‐nKO on the integrity of axonal structure and function in TBI mice.

Furthermore, the sensorimotor behavioral tests showed that the nKO TBI mice outperformed both WT TBI and D‐nKO TBI mice, with no significant differences between the WT TBI and D‐nKO TBI mice (Figure 8F–H), while no significant differences among the three Sham groups (Figure S7C–E, Supporting Information). These results suggest that D‐nKO reverses the sensory‐motor function improvements observed in PTEN‐nKO TBI mice. Furthermore, the Morris Water Maze test revealed that nKO TBI mice significantly outperformed WT TBI and D‐nKO TBI mice in the number of platform crossings, with no significant differences between WT TBI and D‐nKO TBI mice (Figure 8I,J), while there were no significant differences in swimming speeds among the three TBI groups (Figure 8K). These results further confirmed that D‐nKO reversed the long‐term cognitive protective effects of PTEN‐nKO post‐TBI.

Collectively, these results demonstrated that D‐nKO reversed the long‐term protective and reparative effects of PTEN‐nKO on TBI at both behavioral and histological levels, confirming the critical role of the mTORC1 signaling pathway in the neuroprotection mediated by PTEN‐nKO following TBI.

## 3 Discussion

PTEN is extensively expressed in the brains of both humans and mice,^[^ ^46^ ^]^ where it plays a critical role not only in regulating the migration and differentiation of neural cells during development but also in the pathological processes following neuronal injury.^[^ ^47^, ^48^, ^49^ ^]^ Previous studies have reported that PTEN knockout significantly enhances the regenerative capacity of axons after injury,^[^ ^14^, ^50^ ^]^ however, the downstream pathways that are activated following PTEN loss remain incompletely understood. In this study, using neuron‐specific PTEN knockout transgenic mice and neuron‐specific PTEN/Raptor double knockout transgenic mice, we investigated the long‐term roles of PTEN following TBI. Our findings revealed that in neurons lacking PTEN, the Akt/mTOR pathway was continuously activated post‐TBI, persistently enhancing the activation of 4E‐BP1, which promotes protein translation, and elevates the expression of the axon growth‐associated protein GAP43. Consequently, these mechanisms support long‐term axonal regeneration and circuit remodeling. Our results provide evidence that PTEN serves as an important therapeutic target for TBI.

TBI often leads to significant white matter axonal damage, primarily characterized by axonal swelling and breakage, a condition known as diffuse axonal injury (DAI).^[^ ^51^, ^52^ ^]^ This damage disrupts neural circuits and critically affects the prognosis of TBI.^[^ ^53^ ^]^ Effective axonal regeneration is crucial for postinjury synaptogenesis, and synaptic maintenance, and is essential for axonal remapping and functional recovery.^[^ ^54^, ^55^ ^]^ Park and colleagues found that PTEN deletion, by activating the mTOR signaling pathway, significantly enhances the regeneration of injured retinal ganglion cell axons.^[^ ^14^ ^]^ Similarly, enhancement of postinjury regeneration in the corticospinal tract has also been observed with PTEN deletion.^[^ ^24^ ^]^ However, the specific role of the PTEN/mTOR pathway in axonal regeneration and remapping during the chronic phase of TBI remains not well understood. It is still unknown whether PTEN exerts its protective effects during the chronic phase of TBI specifically through the mTORC1 or mTORC2 pathway.

In this study, we investigated whether PTEN deletion could promote neural repair and axonal regeneration over the long term after TBI, along with the underlying mechanisms involved. We observed that the phosphorylation levels of PTEN continued to decrease post‐TBI (Figure 1), indicating that the change was not merely transient. This suggests that PTEN deletion might sustainably influence the activation of the downstream Akt/mTOR pathway, thereby promoting neural regeneration and recovery after TBI. To further verify the long‐term regenerative and reparative effects of PTEN deletion on white matter axons, we employed 9.4T DTI‐MRI live imaging for continuous tracking of white matter fibers (Figure 4). Our results show that PTEN‐nKO provided acute and subacute protection, but chronic‐phase white matter repair (day 35) was significantly more pronounced (Figures 3E and 4E). While our focus on chronic repair, acute‐phase protection may contribute to long‐term recovery. Future studies using tamoxifen‐induced PTEN knockout at 5 days post‐TBI could clarify the relationship between early protection and chronic repair. Additionally, through RNA sequencing and GSEA analysis, we identified significant enrichment in pathways related to the presynaptic cytoskeleton, distal axon, axon terminus, and other aspects critical to axonal and synaptic repair. While PTEN‐nKO significantly promotes axonal regeneration within white matter, it does not provide notable protection or restorative effects on the myelin component, as shown in Figure S4 (Supporting Information). Instead, its primary impact is observed in the axonal structures, as highlighted in Figure 3. This distinction suggests that the therapeutic benefits of PTEN‐nKO are predominantly driven by its influence on axonal recovery rather than on myelin repair, emphasizing its specific role in supporting axonal regeneration after TBI. Notably, we observed a significant enhancement in axonal growth activities, as evidenced by increased expression of GAP43. Interestingly, the RNA‐seq data did not show upregulation of the GAP43 gene, likely because the RNA sequencing was conducted during the acute post‐TBI period, while GAP43 protein levels were assessed 35 days after TBI. This discrepancy suggests that GAP43 may play a more pronounced role in nerve regeneration and repair in the long term following TBI. Furthermore, we successfully validated the improvement of sensory upward pathways and motor downward pathways after TBI using fiber photometry recordings of bulk neuronal activity and electromyographic recordings (Figure 5), which facilitated a deeper understanding of the connectivity remodeling in the somatosensory motor cortical neural circuits post‐injury. Previous studies have shown that PTEN deletion promotes axonal regeneration primarily by enhancing mTOR activation, which in turn activates downstream molecules 4E‐BP1.^[^ ^49^, ^50^ ^]^ In line with these findings, we found that neuronal PTEN knockout post‐TBI activates the Akt/mTOR/4E‐BP1 pathway (Figure 6). Collectively, these experimental results support our hypothesis that during the chronic recovery phase post‐TBI, PTEN deletion can promote long‐term axonal regeneration/remapping and facilitate the remodeling and recovery of neural circuit functions through activation of the Akt/mTOR pathway.

In this study, we confirmed that PTEN deletion during the chronic recovery phase of TBI promoted activation of the mTORC1/4E‐BP1 axis (Figure 6) and observed an upregulation of GAP43 expression (Figure 4). However, whether mTORC1/4E‐BP1 directly upregulates GAP43 remains inconclusive. It remains unclear whether phosphorylated 4E‐BP1 regulates GAP43 directly, or indirectly through modulation of transcription factors such as NF‐κB or STAT3, which are known to upregulate GAP43 expression.^[^ ^56^ ^]^ Future research could investigate whether 4E‐BP1 directly or indirectly regulates GAP43 expression.

In our detailed investigation of downstream signaling mechanisms, we demonstrate that mTORC1 plays a crucial role in how PTEN promotes long‐term axonal regeneration and cortical remapping post‐TBI. We generated a double neuron‐specific knockout transgenic mouse model for PTEN/Raptor (Figure 7), where Raptor is a crucial component for the stability and functionality of mTORC1.^[^ ^43^, ^57^ ^]^ By utilizing this model, we could inhibit the activation of mTORC1 and its downstream pathways simultaneously with neuronal PTEN deletion. We then performed long‐term assessments of white matter axonal recovery, as well as comprehensive histological, somatosensory motor, and learning and memory behavioral tests (Figures 7 and 8) on the D‐nKO mice. These assessments consistently demonstrated that D‐nKO mice almost completely reversed the long‐term protective and reparative effects of PTEN deletion on TBI. From these results, we concluded that PTEN deletion promotes long‐term regeneration and repair of white matter axons post‐TBI predominantly through mTORC1.

Our study has some limitations. In this study, we demonstrated that PTEN deletion aids long‐term white matter axon regeneration and repair after TBI. However, PTEN inhibition might also affect the acute phase post‐TBI, potentially involving mechanisms such as ferroptosis and neuroinflammation, which warrants further research. Additionally, further investigation is needed to explore PTEN's role in white matter damage through other mechanisms and its function in other neural cells, such as microglia and astrocytes, after TBI. In this study, we demonstrated the dependency of PTEN‐nKO‐induced axonal regeneration on mTORC1 activity using PTEN/Raptor D‐nKO mice. While these results establish a critical link, future studies utilizing neuronal mTORC1 knockout or pharmacological mTORC1 inhibitors could further validate this mechanism. Such complementary approaches would provide additional evidence supporting the causal role of mTORC1 in mediating the therapeutic effects of PTEN‐nKO and offer deeper insights into the spatial and temporal dynamics of mTORC1 modulation during TBI recovery. Furthermore, the application of PTEN knockout is not feasible for clinical translation, highlighting the need for PTEN inhibitors suitable for treating TBI. Currently, PTEN inhibitors such as VO‐OHpic and SF1670 are available, but their efficacy in targeting the nervous system is limited. Therefore, there is a critical need to design PTEN inhibitors with specificity for the neural system, particularly those that target neurons.^[^ ^58^, ^59^ ^]^

## 4 Conclusion

In summary, our study demonstrated that PTEN is persistently activated after TBI, and that neuronal PTEN deletion can sustainably activate the Akt‐mTORC1 pathway, promoting long‐term neural regeneration and neural circuit remodeling, ultimately leading to improved outcomes. These findings advance our understanding of white matter axonal regeneration and provide potential therapeutic insights for improving recovery following TBI.

## 5 Experimental Section

### Experimental Animals

PTEN^flox/flox^ mice (B6.129S4‐Ptentm1Hwu/J) were purchased from the Jackson Laboratory. MAP2^CreERT2^ mice (C57‐BL/6‐Map2em1(2A‐CreERT2)Smoc) and Raptor^flox/wt^ mice (Rptor‐eCKO1) were purchased from Shanghai Southern Model Biotechnology Development Co. Neuron‐specific PTEN knockout (PTEN‐nKO) mice were obtained by crossing PTEN^flox/flox^ mice and MAP2^CreERT2^ mice, and neuron‐specific Raptor/PTEN double‐knockout mice (D‐nKO) were obtained by crossing Raptor^flox/flox^ mice with PTEN‐nKO mice. Both PTEN‐nKO mice and D‐nKO mice (10–12‐week‐old) were injected intraperitoneally with tamoxifen (100 mg kg^−1^ for 5 days) to induce neuron‐specific knockouts of PTEN or both PTEN and Raptor. Heterozygous mice (genotype: PTEN^flox/flox^⋅MAP2^CreERT2−/−^ or Raptor^flox/flox^⋅PTEN^flox/flox^⋅MAP2^CreERT2−/−^) were used as wild‐type (WT) control mice, which were also subjected to tamoxifen injection. The mice were injected with tamoxifen and then subjected to controlled cortical impingement (CCI) after 10 days.

### TBI Model

The TBI model was established using the CCI technique with a device (TBI 0310, Precision Systems and Instrumentation), as described in detail in a previous study.^[^ ^18^, ^28^ ^]^ In brief, the mice were anesthetized using 1–2% isoflurane mixed with 70% N2/30% O2 and placed on the stereotaxic apparatus. A cranial window was then formed over the right temporal lobe. A cranial window was then created over the right temporal lobe. The CCI was conducted at centered coordinates (AP: −0.5, ML: 2.0) with a 30‐mm flat‐tip impounder (velocity, 3.5 ms^−1^; duration, 150 ms; depth, 1.5 mm). All these procedures were applied to sham mice except for the CCI.

### Neurobehavioral Testing

In this study, sensory‐motor dysfunctions in mice were evaluated using the Neurological Severity Score (NSS),^[^ ^60^ ^]^ Grid‐Walking Test,^[^ ^61^ ^]^ and Wire‐Hanging Test^[^ ^13^ ^]^ at 3, 5, 7, 14, 21, 28, and 35 days post‐TBI.^[^ ^28^ ^]^ A Morris water maze test was conducted from 29 to 34 days after a traumatic brain injury to evaluate long‐term cognitive deficits.^[^ ^28^, ^62^, ^63^ ^]^

### MRI Measurement

An in vivo MRI scan was performed using a high‐field‐strength 9.4T small‐animal MRI system (United Image, Shanghai, China) at 3,10, and 14 days post‐TBI.^[^ ^64^, ^65^ ^]^ MRI scanning was performed on mice anesthetized with 3% isoflurane and maintained with 1% isoflurane in the coil. Structural changes in the brain tissue after TBI, including brain tissue damage and hydrocephalus, were meticulously assessed using T2‐weighted imaging (T2WI) sequences. The T2WI scan was carried out with the following parameters: field of view (FOV) = 18 × 18 mm, repetition time (TR)/echo time (TE) = 3000/26 ms, acquisition matrix = 256 × 256, 45 slices, slice thickness = 0.3 mm, averaging two measurements, Relaxation Enhancement (RARE) factor = 8.

To assess the cerebral white matter microstructure, a Diffusion Tensor Imaging (DTI) scan was performed. After saline and paraformaldehyde perfusion, the mice's brains were fixed in PFA for 24 h. The brains were then air‐dried and subsequently placed in test tubes filled with carbon‐free oil, then scanned with the machine's coils. For the ex vivo T2WI scan, the following parameters were chosen: FOV = 20 × 20 mm, TR/TE = 4000/17.331636364 ms, acquisition matrix = 256 × 256, 28 slices, slice thickness = 0.4 mm, averaging two measurements, RARE factor = 8.

### Bulk RNA Sequencing Analysis

The data quality was assessed using FastQC (version 0.11.9). Low‐quality reads (with a quality score below 20) were removed using Trim Galore. Reads were aligned to mm10 (Mus musculus, UCSC mm10) using HISAT2 (version 2.2.1) after being sorted using Samtools. Mapped reads were quantified using HTSeq (version 1.99.2) for each gene annotated in the genome reference GRCm38.p6 (GENCODE release M25). DESeq2 package (version 1.40.2) was used to normalize the raw counts and perform principal components analysis (PCA). Shrinkage of effect sizes using the adaptive shrinkage estimator (ashr) algorithm was applied for visualization and ranking of genes. Genes with an adjusted *p*‐value <0.05 and an absolute log2 fold change >0.58 were defined as differentially expressed genes (DEGs). EnhancedVolcano (version 1.13.2) was employed to visualize the differential expression analysis results. GSEA analysis based on the GO‐Cellular Component (GO‐CC) ontology dataset was performed using the gseGO function from the cluster Profiler (version 4.8.2) to identify enriched terms in the nKO‐TBI versus WT‐TBI comparison.

### Composite Action Potentials (CAPs) Measurement

To assess nerve conduction function after TBI, CAPs in the external capsule were recorded as previously described.^[^ ^13^, ^66^ ^]^ Briefly, following the decapitation of the mouse, the brain was removed and sliced with a vibratome (Leica) into 350m‐thick slices. The slices with damage to peripheral brain areas (Bregma +1.1 mm & −2.1 mm) were collected and then transferred to artificial cerebrospinal fluid (aCSF) (124 mmol L^−1^ NaCl, 2.5 mmol L^−1^ KCl, 2 mmol L^−1^ CaCl_2_, 1 mmol L^−1^ NaH_2_PO_4_, 24 mmol L^−1^ NaHCO_3_, 1.3 mmol L^−1^ MgSO_4_, and 10 mmol L^−1^ D‐glucose). Subsequently, slices were incubated in oxygenated aCSF with a 95% O2/5% CO2 mixture for 0.5 h at 32 °C, followed by 1 h at room temperature. CAPs were induced using a bipolar stimulation generator (model STG 4002, Multi‐Channel Systems) by applying a series of monophasic square‐wave stimuli (0.1 ms duration) to the corpus callosum at ≈0.1 mm deep and 0.9 mm lateral to the midline, with the current starting at 0 mA and increasing by 0.25 mA each time up to a maximum of 2 mA. The evoked CAPs were recorded using glass microelectrodes filled with aCSF with a resistance of 5–8 MΩ at the EC position1 mm from the stimulation electrode. The Axoclamp 700B amplifier (Molecular Devices) amplification and Axon Digidata 1440A (Molecular Devices) digitization were used to record the signals.

### Fiber Photometry Recording and Data Analysis

Two weeks after TBI surgery, mice were anesthetized with 1–2% isoflurane mixed with 70% N2/30% O2 and positioned in a stereotaxic injection apparatus (68037, RWD). A total of 300 nL of AAV2/9‐hSyn‐GCaMP6m‐WPRE‐pA (Shanghai Taitool Bioscience, Shanghai, China, 1.012 × 10^12 V.G. mL^−1^, S0471‐9) was injected into the contralateral cortex [bregma: AP = 0.5 mm, ML = 2.0 mm, DV = 1.7 mm; forelimb first sensory zone (S1FL)] using a micro syringe pump controller at a rate of 30 nL min^−1^ (53311, Stoelting). After injection, the pipette remained in place for 10 min before being withdrawn, and then an optic fiber (200 µm; BGFLaser, Xi'an) was implanted 0.2 mm above the injection site and secured with dental cement. Fiber photometry recording experiments were performed three weeks after recovery.

For fiber photometry recording, experiments were conducted using a fiber photometry system (Thinker Biotech, Nanjing). During all recording experiments, the light intensity of the 470 nm channel was adjusted to 30 µW, and calcium data from tactile stimulation (100 Hz vibration stimulus) of both forepaws were collected. The calcium signal data were then exported to MATLAB for further analysis. The fluorescence change (ΔF/F) was calculated using the formula (F–F0)/F0, where F0 was the mean baseline fluorescence (−5 to 0 s), and the data were z‐scored for each trial (both paws). Finally, the mean ΔF/F (z‐score) from 0 to 2 s was used for statistical analysis.^[^ ^13^ ^]^

### EMG Recording and Data Analysis

For the EMG surgery, mice were anesthetized with 1–2% isoflurane mixed with 70% N2/30% O2 and placed on a thermostatic heating plate set at 37 °C. Two metal recording electrodes were carefully implanted into the biceps brachii muscle of the forelimb to record EMG activity, and a reference electrode was inserted ≈3 mm into the forepaw walking pad at a 30° angle. The grounding electrode was placed subcutaneously on one side of the abdominal region. All electrodes were properly separated to avoid signal interference.

For EMG recording, EMG signal data were collected using a three‐channel EEG/EMG recording system (Pinnacle Technology 8206‐HR). EMG signals from tactile stimulation (100 Hz vibration stimulus and 10 g weight compression) of both forelimbs were collected. The mean difference between the maximum and minimum amplitudes of ten stimulus trials was used for quantitative analysis.

### Immunohistochemistry

After ice‐cold saline perfusion and 4% paraformaldehyde immersion, the brains were then removed and sequentially immersed in 4% paraformaldehyde, 20% sucrose, and 30% sucrose to complete the postfixation and dehydration process. Subsequently, 25 µm coronal slices were obtained using a cryostat. Following the washing process with PBS, blocking with 5% donkey serum, and incubation with primary antibody overnight at 4 °C, the selected brain slices were incubated with secondary antibody for an hour, followed by mounting with DAPI Fluoromount‐G. The images of stained brain slices were captured by a Nikon A1 confocal microscope and analyzed by ImageJ software. Primary antibodies used included rabbit anti‐NeuN (ab104225, Abcam, USA), rabbit anti‐NF200 (ab8135, Abcam, USA), rat anti‐MBP (ab7349, Abcam, USA) rabbit anti‐Synaptophysin (ab32127, Abcam, USA), mouse anti‐PSD95(#36233, Cell signaling Technology, USA). All the fluorescence secondary antibodies were purchased from Jackson ImmunoResearch (USA).

### Western Blot

Brain tissue was removed from the periphery of the injury immediately after the animals were executed. The total protein was extracted using cold RIPA buffer (Shanghai Beyotime, China) supplemented with a protease inhibitor cocktail (Roche). Equivalent amounts of extracted proteins (30 µg) were separated by SDS‐PAGE and transferred to PVDF membranes. PVDF membranes were blocked with 5% BSA for 1.5 h and then incubated overnight at 4 °C with primary antibodies and secondary antibodies the following day for 1 h at room temperature. Primary antibodies used included rabbit anti‐Phospho‐PTEN (44‐1066G, Invitrogen, USA), rabbit anti‐PTEN (9559, Cell signaling Technology, USA), rabbit anti‐p‐Akt (2118‐1, Epitomics, USA), rabbit anti‐Akt (4691, Cell signaling Technology, USA), rabbit anti‐p‐4E‐BP1 (2855, Cell signaling Technology, USA), rabbit anti‐4E‐BP1 (9644, Cell signaling Technology, USA), rabbit anti‐p‐mTOR (5536, Cell signaling Technology, USA), rabbit anti‐mTOR (2983, Cell signaling Technology, USA), rabbit anti‐Raptor(#2280, Cell signaling Technology, USA), rabbit anti‐GAP43 (8945, Cell signaling Technology, USA). The secondary antibodies were goat anti‐mouse/anti‐rabbit IgG conjugated with HRP (Cell Signaling Technology, USA). An ECL chemiluminescence development system was applied to develop the images, and images were captured with a Bio‐rad ChemiDoc developer (Bio‐Rad, USA). Quantitative protein analysis was performed by ImageJ software, normalizing target protein expression levels to β‐actinexpression levels.

### Statistical Analysis

Data were analyzed using SPSS Statistics 20 and GraphPad Prism 10. All data are presented as Means ± Standard Deviation (Means±SD). The Kolmogorov‐Smirnov test was used to assess the normality of all datasets. For normally distributed data, differences between groups were analyzed using one‐way or two‐way ANOVA, followed by Bonferroni post‐hoc tests for multiple comparisons. Non‐normally distributed data were analyzed using non‐parametric methods: the Wilcoxon signed‐rank test for two independent samples and the Kruskal‐Wallis test for three or more independent samples. Differences between cumulative curves were assessed using the Kolmogorov‐Smirnov test. The unpaired two‐tail Student's *t*‐test was used for comparisons between two independent groups. Spearman correlation analyses were used to explore associations between different variables. *p* < 0.05 were considered statistically significant.

### Ethics Statement

Animals were housed in environments with a 12‐h light‐dark cycle, suitable temperature and humidity, and free access to food and water. All animal procedures were approved by the Experimental Animal Ethics Committee, Fudan University (approval number: 2018‐JS‐003 and 20240229‐143) and reported following the ARRIVE guidelines. The study minimized animal suffering and used a minimum number of animals. Informed consent was obtained from human participants before tissues were collected. Patients’ tissue was obtained and used in a manner compliant with the Declaration of Helsinki and all procedures were approved by the Ethics Committee of Huashan Hospital affiliated with Fudan University (Approval Number: 2023–095).

## Conflict of Interest

The authors declare no conflict of interest.

## Author Contributions

Z.S., L.M., and S.C. contributed equally to this work. Y.G. designed the study. Z.S., L.M., S.C., J.X., M.S., Y.W., Y.W., X.C. performed the experiments. Z.S. analyzed the data, L.M. checked up the data. Z.S. and L.M. wrote the manuscript. Y.G. and Z.X. critically edited the manuscript. Z.D. collected the patient's samples. The authors thank the patients for the donation of the tissue samples. Z.S., L.M., and S.C. contributed equally to this study. The authors read and approved the final manuscript.

## Supporting information



Supplementary Figures

## Data Availability

The data that support the findings of this study are available from the corresponding author upon reasonable request.
