# Supplementary material for: Reversing Persistent PTEN Activation after Traumatic Brain Injury Fuels Long‐Term Axonal Regeneration via Akt/mTORC1 Signaling Cascade
Source: Adv Sci (Weinh). 2024 Dec 16;12(6):2410136. doi: 10.1002/advs.202410136 (PMC11809353; doi:10.1002/advs.202410136)
Supplement: Supplementary file 1 — Supplementary Figures [file ADVS-12-2410136-s001.pdf]

ADVANCED SCIENCE
Open Access

Supporting Information

for *Adv. Sci.*, DOI 10.1002/advs.202410136

Reversing Persistent PTEN Activation after Traumatic Brain Injury Fuels Long-Term Axonal Regeneration via Akt/mTORC1 Signaling Cascade

Ziyu Shi, Leilei Mao, Shuning Chen, Zhuoying Du, Jiakun Xiang, Minghong Shi, Yana Wang, Yuqing Wang, Xingdong Chen, Zhi-Xiang Xu and Yanqin Gao**

Supporting Information

Unlocking Neural Limitation: How Reversing Persistent PTEN Activation After Traumatic Brain Injury Fuels Long-Term Axonal Regeneration/Remapping via the Akt/mTORC1 Axis

Ziyu Shi[#1], Leilei Mao[#1], Shuning Chen[#1], Zhuoying Du[2], Jiakun Xiang[1], Minghong Shi[1], Yana Wang[1], Yuqing Wang[1], Xingdong Chen[1], Zhi-Xiang Xu*[1] and Yanqin Gao*[1]

[1]State Key Laboratory of Medical Neurobiology, MOE Frontiers Center for Brain Science, and Institutes of Brain Science, Fudan University, Shanghai, China

[2]Department of Neurosurgery, Huashan Hospital, Fudan University, Shanghai, China

# The authors contributed equally to this study.

Corresponding authors: Yanqin Gao (yqgao@shmu.edu.cn) or Zhi-Xiang Xu (zhixiangxu@fudan.edu.cn)

Supplementary Figures

A

Patient details

Patient ID	Age	Gender	Diagnosis	Tissue position	GCS
1	43	M	Brain contusion	left temporal lobe	8
2	56	M	Brain contusion	left frontal lobe	11
3	51	F	Brain contusion	left temporal lobe	5
4	68	F	Brain contusion	right temporal lobe	12

GCS: Glasgow Coma Scale

B

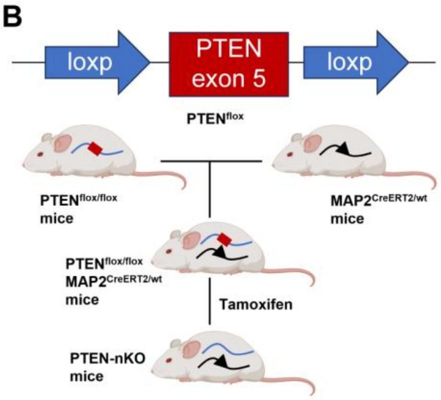

Figure S1. Cerebral contusion patient information and PTEN-nKO mouse construction. (A) The information of the four patients with acute cerebral contusion is shown in Table 1, and all of them underwent emergency surgery at the Department of Neurosurgery, Huashan Hospital, Shanghai, China. (B) Schematic diagram of PTEN-nKO mouse construction.

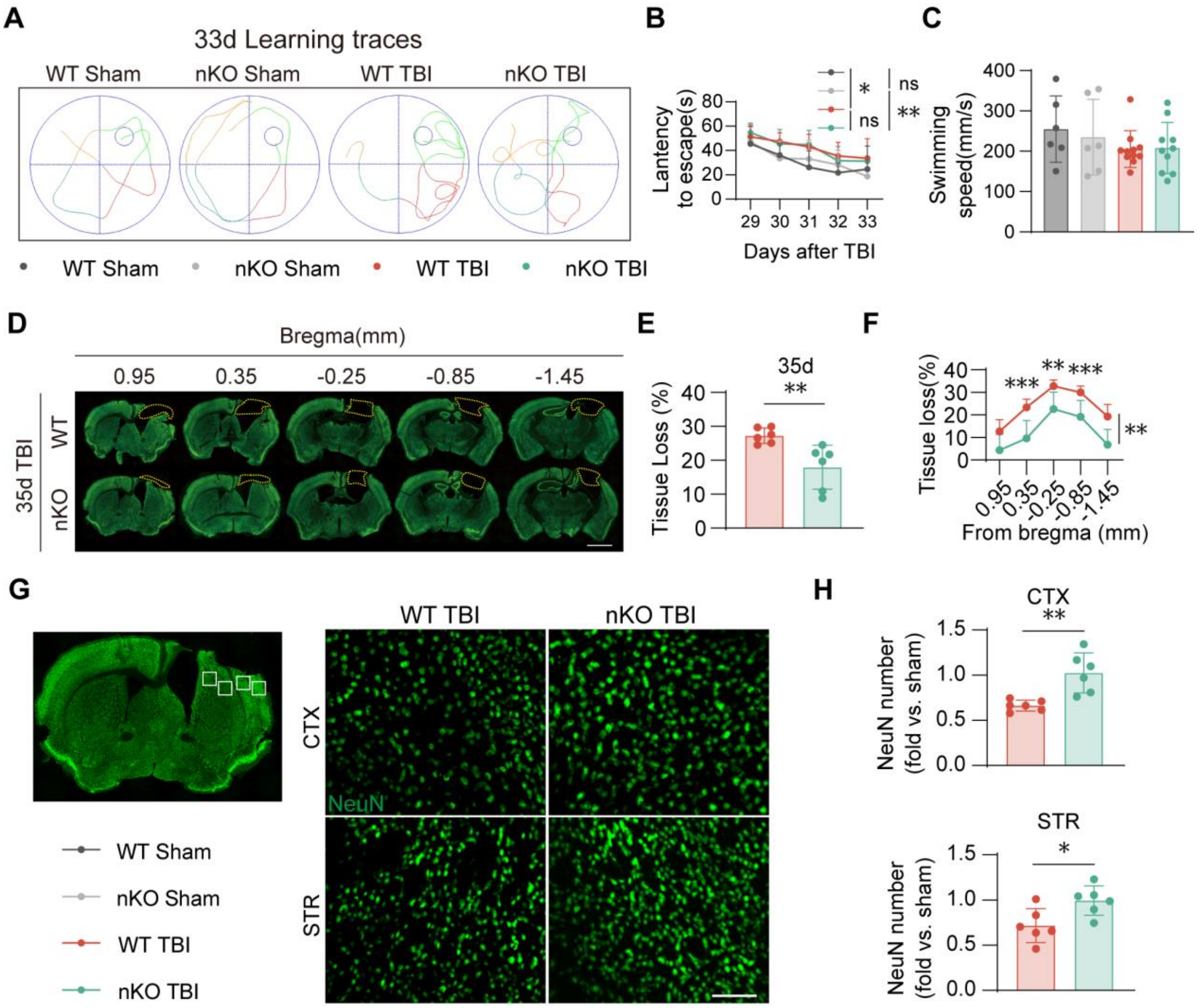

Figure S2. Water Maze Learning Test and NeuN Staining Results in the long-term after TBI. (A&B) Representative swimming paths (A) and quatification of lantency time (B) during the learning phases of the Morris water maze test. (C) Quantification of swimming speed during the memory phases of the Morris water maze test (34 days post-TBI). n=6/6/11/10 (WT Sham/nKO Sham/WT TBI/nKO TBI). (D) Representative images of NeuN staining (green) for brain sections 35 days post-TBI, bar: 1mm. (E&F) Quantification of the tissue loss volume (E) and the area (F) from bregma 0.95 mm to -1.7 mm on day 35 post-TBI, n=6/group. (G) Representative images of NeuN staining in the peri-TBI area 35 days post-TBI. Bar: 100μm. (H) Quantification of neuron number in the CTX and STR of the peri-TBI area 35 days post-TBI, n=6/group. All data are presented as the means±SD. Data were analyzed using one-way (C) or two-way (B, F) ANOVA followed by Bonferroni's post hoc test, or unpaired with two-tails Student's t-test (E, H). **p*<0.05, ***p*<0.01, ****p*<0.001, ns: no significance, as indicated.

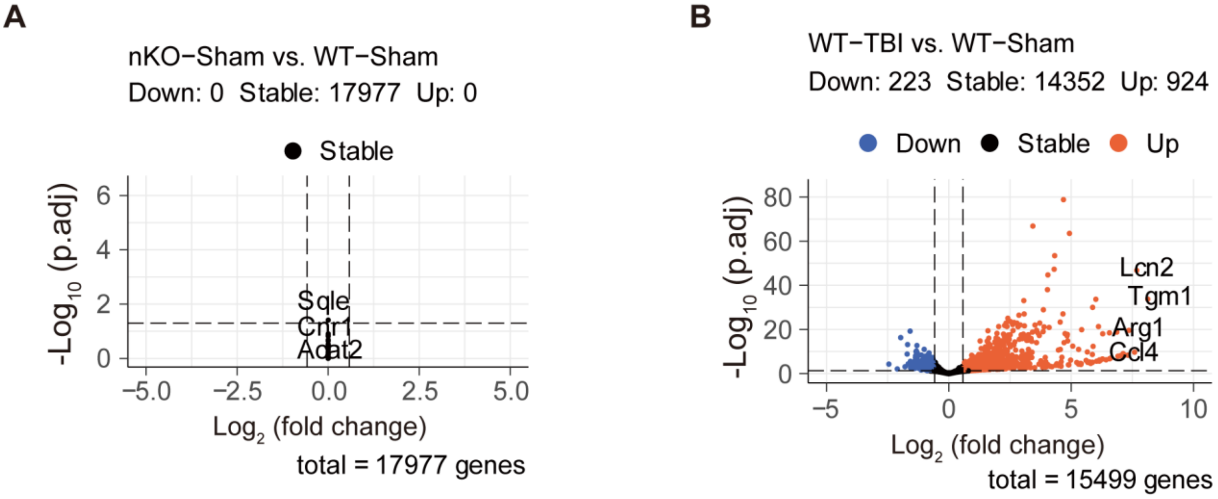

Figure S3. Bulk RNA-seq 1 day after TBI. (A) Volcano plot of differential expression genes for WT- Sham *vs.* nKO- Sham. (B) Volcano plot of differential expression genes for WT TBI *vs.* WT Sham.

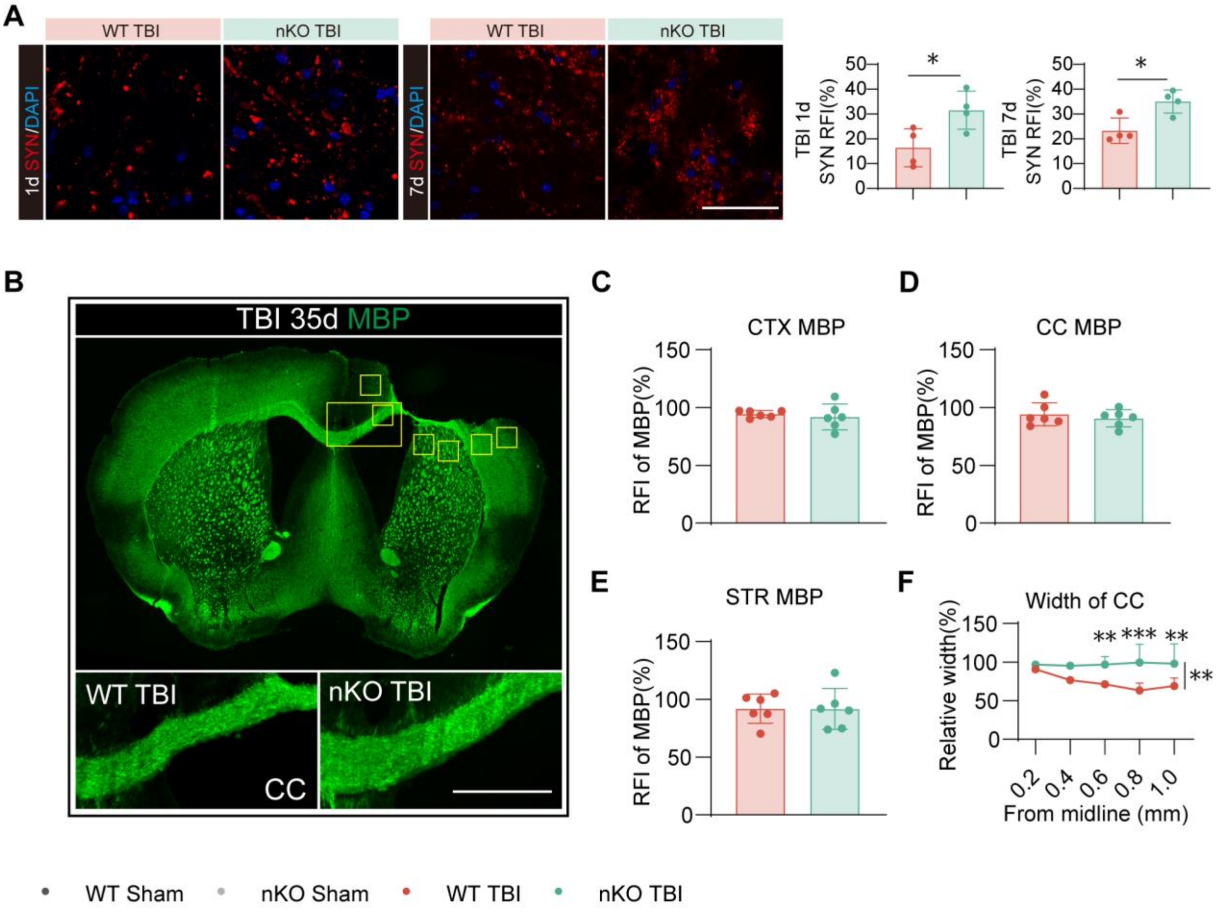

Figure S4. White matter damage after TBI. (A) Representative images and statistical plots of synaptophysin (red) and DAPI (blue) immunofluorescence in the peri-injury zone of the CTX at 1

day and 7 days post-TBI, bar: 50 μm. n=4/group. (B) Representative images of MBP staining (green) 35 days post-TBI. (C-E) Quantification of MBP RFI in the CTX, STR, and CC of the peri-TBI area. n=6/group. (F) Quantification of the relative width of CC in the ipsilateral. n=6/group. All data are presented as the mean±SD, Data were analyzed using unpaired with two-tails Student's t-test (A, C-E), one-way ANOVA followed by Bonferroni's post hoc test (F). *p<0.05, **p<0.01, ***p<0.001, as indicated.

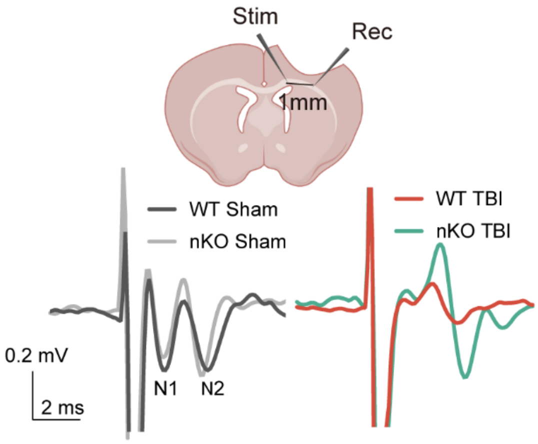

Figure S5. Schematic representation of a composite action potential (CAP) recording from the corpus callosum.

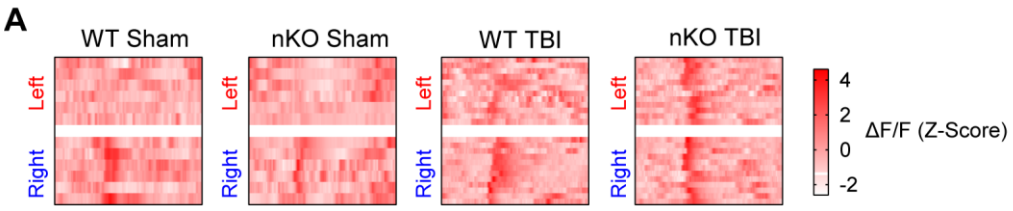

Figure S6. Heatmap displaying calcium signal changes (A) Heatmap displaying calcium signal changes in response to vibratory stimulation of both forelimbs, detected by fiber optics in the S1FL region 35 days post-TBI. The time range is from 5s before to 10s after stimulation, with red indicating the left forelimb with impaired function and blue indicating the right forelimb with normal function. Sham group: n=2 animals × 3 times; TBI group: n=4 animals × 3 times.

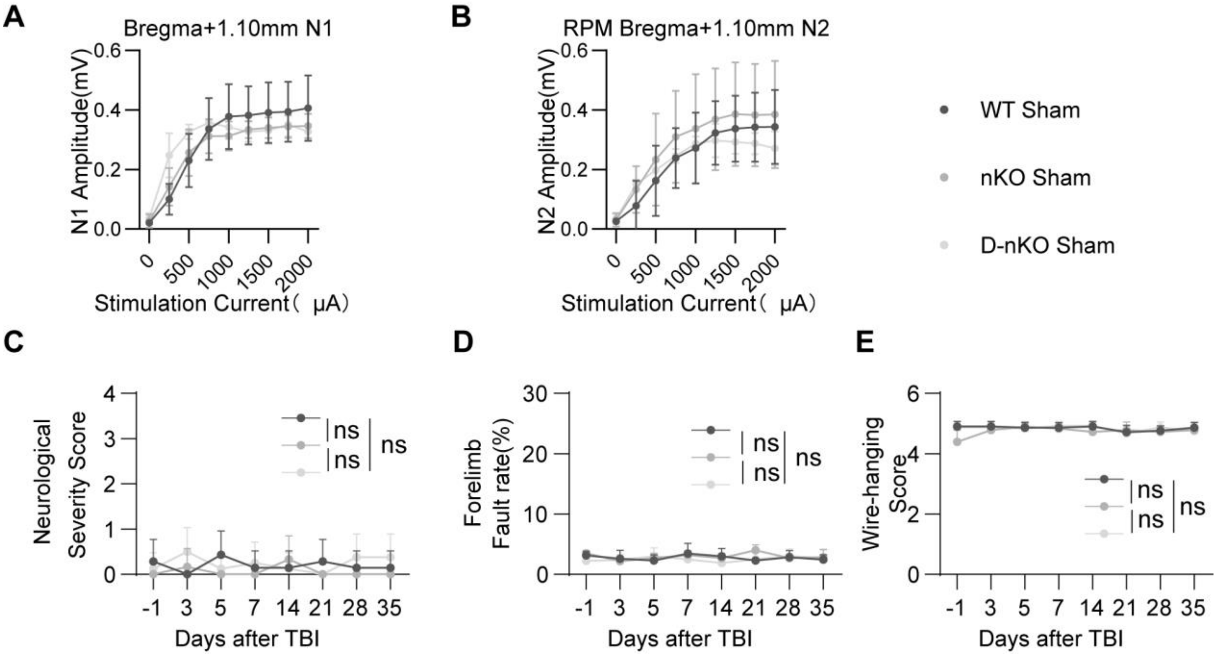

Figure S7. PTEN/Raptor D-nKO and PTEN-nKO did not affect CAP and behavioral performance in the Sham group. (A&B) Statistical plots showing the N1 and N2 amplitudes for bregma+1.10 mm in CC on day 35 post-TBI. n=5/4/2 (WT Sham/nKO Sham/D-nKO Sham). (C-E) Sensorimotor deficits were evaluated before (-1) and up to 35 days after TBI by neurological severity scores (C), the grid walking test (D), and the wire-hanging test (E). C-E are presented as means±SD, n=7/6/8 (WT Sham/nKO Sham/D-nKO Sham). Data were analyzed using two-way ANOVA followed by Bonferroni post hoc test. ns: no significance, as indicated.
